# Physiological Signals and Affect as Predictors of Advertising Engagement

**DOI:** 10.3390/s23156916

**Published:** 2023-08-03

**Authors:** Gregor Strle, Andrej Košir, Urban Burnik

**Affiliations:** 1 User-Adapted Communication and Ambient Intelligence Lab, Faculty of Electrical Engineering, University of Ljubljana, SI 1000 Ljubljana, Slovenia; andrej.kosir@fe.uni-lj.si (A.K.); urban.burnik@fe.uni-lj.si (U.B.); 2 Scientific Research Centre, ZRC SAZU, SI 1000 Ljubljana, Slovenia

**Keywords:** physiology, affect, engagement, advertisement, user modeling, classification, machine learning

## Abstract

This study investigated the use of affect and physiological signals of heart rate, electrodermal activity, pupil dilation, and skin temperature to classify advertising engagement. The ground truth for the affective and behavioral aspects of ad engagement was collected from 53 young adults using the User Engagement Scale. Three gradient-boosting classifiers, LightGBM (LGBM), HistGradientBoostingClassifier (HGBC), and XGBoost (XGB), were used along with signal fusion to evaluate the performance of different signal combinations as predictors of engagement. The classifiers trained on the fusion of skin temperature, valence, and tiredness (features *n* = 5) performed better than those trained on all signals (features n = 30). The average AUC ROC scores for the fusion set were XGB = 0.68 (0.10), LGBM = 0.69 (0.07), and HGBC = 0.70 (0.11), compared to the lower scores for the set of all signals (XGB = 0.65 (0.11), LGBM = 0.66 (0.11), HGBC = 0.64 (0.10)). The results also show that the signal fusion set based on skin temperature outperforms the fusion sets of the other three signals. The main finding of this study is the role of specific physiological signals and how their fusion aids in more effective modeling of ad engagement while reducing the number of features.

## 1. Introduction

Advances in technology and digital media advertising have enabled new approaches to measuring consumer engagement and exposure to online advertisements (ads) [1]. One area of advertising that is growing rapidly is ad-supported video streaming, which has overtaken video-on-demand streaming [2]). One advantage of these digital advertising platforms is that viewers’ responses to advertising can be measured directly and continuously [3]. This is now possible with unobtrusive, wearable sensing devices, such as smartwatches, that have a plethora of sensors, including accelerometers, gyroscopes, heart rate, blood flow, electrodermal activity, and skin temperature. Video-on-demand advertising would benefit from such wearable technology as it provides quick and accurate insights into consumer engagement and exposure to ads based on physiological signals.

Advertising evokes emotional responses and triggers cognitive processes in consumers [4,5]. This, in turn, influences individuals’ physiological responses and can provide new insights into consumer behavior. Owing to the steady development of wearable devices with physiological sensors, physiological signals may provide a way to directly and continuously measure the effects of ad exposure and better understand consumer behavior [1].

This study investigated whether physiological signals could be used as a measure of emotional engagement in video advertising. Emotional engagement is defined as “the amount of sub-conscious “feeling” going on when an advertisement is being processed” [6] (p. 67). It is one of the key aspects of understanding engagement in ads, as elevated arousal has been shown to increase engagement behavior [7]. We hypothesized that physiological signals, known to be associated with emotional arousal and valence [8,9], could provide a reliable and unobtrusive measure of ad engagement.

To this end, an observational study was conducted to assess the participants’ physiological and affective responses to video ads. The focus group comprised younger adults who use streaming services extensively and are accustomed to in-video ads [3]. The ground truth for ad engagement was collected using the User Engagement Scale-Short Form (UES-SF) [10], an established psychometric instrument for measuring the affective and behavioral aspects of engagement. Physiological signals of heart rate, electrodermal activity, pupil dilation, and skin temperature were recorded as responses to video ads, along with measures of affect. Machine learning was used to model ad engagement as a classification problem, and physiological and affective responses to ads were examined as predictors of lower and higher engagement, respectively.

The presented study contributes to the existing body of knowledge by highlighting the potential of machine learning and signal fusion to improve emotional ad engagement evaluation. The main contributions of this study are as follows: (1) it demonstrates which physiological signals and their features are effective predictors of ad engagement; (2) it shows that the process of signal fusion can maintain classification performance while reducing the number of features; and (3) it shows that predictive modeling works best when signal fusion is employed.

In the following section, we first present related work. In Section 3, the materials and methods used in the experimental study are presented. Details of the experimental design, psychometric and physiological measurements, signal processing, and statistical and machine learning tools and procedures are provided. The results of the statistical analysis, signal fusion, and classifier evaluation are presented in Section 4. The article concludes with a brief discussion of the results and possible directions for future research in Section 5.

## 2. Related Work

### 2.1. Ad Engagement

Engagement is a multidimensional concept, covering the affective, behavioral, and cognitive aspects of individuals’ interactions with advertisements and brands [1,10,11,12,13,14]. According to Hollebeek et al, customer engagement is “a customer’s motivationally driven, volitional investment of operant resources (including cognitive, emotional, behavioral, and social knowledge and skills), and operand resources (e.g., equipment) into brand interactions” [15]. Higher consumer engagement leads to more attention, improves brand memorability and attitude [16,17], and can positively influence consumer purchase intentions [1,13]. Therefore, consumer engagement is an important metric to quantify brand exposure and advertising effectiveness.

An analysis of over 200 media exposure studies conducted by [18] found that there is no one-size-fits-all solution for defining and measuring engagement because consumer engagement is based on context-specific experiences and attitudes. In the digital advertising landscape, novel approaches to measuring ad engagement that focus on the affective and behavioral aspects of consumers’ interactions with ads are increasingly being considered [1,5,11].

For example, [12] examined engagement in terms of behavioral manifestations in the consumer–brand relationship, while [5,11] focused on affective responses to ads. The latter are key to understanding ad engagement, as elevated arousal has been shown to increase engagement behavior [7]. To this end, Eijlers et al. [5] examined how arousal is represented in the brain in response to ads, measured by notability and attitude toward ads. An electroencephalogram (EEG) was recorded for 23 female participants (age: 18–30 years). Their results showed that arousal was positively associated with notability, but attitude toward the ads was negatively associated with arousal in response to the ads. The behavioral and social dimensions of engagement were conceptualized using the User Engagement Scale (UES) developed in [10]. UES measures affect, aesthetic appeal, focused attention, novelty, perceived usability, felt involvement, and endurability [10].

Most of these studies have examined ad engagement using self-reported data alone. This has several drawbacks: self-reporting is time-consuming and prone to rater bias [19]. Also, it cannot measure ad engagement directly. In the context of ad-supported video streaming, self-reports are difficult to implement because they distract consumers from immersive experience [16]. All of these shortcomings were the main motivations for the alternative presented here: to investigate direct and unobtrusive measures of ad engagement.

### 2.2. Physiological Measurement of Engagement

More direct methods of measuring consumer engagement are being explored, with the goal of measuring ad engagement unobtrusively and in real time, without interrupting the streaming experience.

One approach is to measure ad engagement using psychophysiological signals associated with emotional arousal and cognitive processes. Physiological signals have been studied in detail as potential indicators of a user’s emotional arousal and cognitive load in various human–computer interaction situations. Signals such as electroencephalography, cardiac (heart rate and heart rate variability), electrodermal activity, skin temperature, respiratory activity, and eye measurements (e.g., gaze, pupil dilation, eye blinking) have all been found to be associated with emotional arousal and cognitive load [20,21,22,23].

A study conducted by [24] demonstrated that common physiological signals such as heart rate, skin temperature, respiratory rate, oxygen saturation, blood pressure, and electrocardiogram (ECG) data can be used to measure engagement. Another study [25] examined engagement during video and audio narration using wrist sensors for heart rate variability, electrodermal activity, and body temperature. There was a significant physiological response to all three measures. Ayres et al. conducted a comprehensive examination of physiological data on intrinsic cognitive load, including pupil dilation, blink rate, fixation, heart rate, heart rate variability, electrodermal measurements, respiratory measurements, functional near-infrared spectroscopy, electroencephalography, and functional magnetic resonance imaging [26]. They found that the blink rate, heart rate, pupil dilation, and alpha waves were the most sensitive physiological measurements.

Pupil dilation and heart rate have been studied extensively as physiological measures of arousal and identified as potential indicators of cognitive performance in participants [9,21,23,27]. In [28], it was suggested that personalized advertising systems based on instantaneous measurement of heart rate variability could be used in future advertising strategies. According to [9], heart rate variability (HRV) can be a reliable and cost-effective source of data for neurophysiological and psychophysiological studies, when appropriate acquisition protocols and well-developed indices are available. Schaffer et al. [29] studied the complexity of cardiac oscillations in their evaluation of methods for measuring HRV in the time and frequency domains, using nonlinear metrics.

Pupil dilation can be used to assess cognitive load. According to [8], an increase in task demands leads to an increase in pupil dilation in the cognitive control domains of updating, switching, and inhibition. However, the study did not provide a clear explanation of the relationship between pupil dilation and performance. Since the early 1970s, researchers have used pupil dilation in studies of advertising effectiveness [30]. As pupil dilation is sensitive to variations in brightness, it is difficult to use pupil dilation to measure emotional arousal during video viewing. In [31], a linear model that predicts a viewer’s pupil diameter based only on the incident light intensity was presented. The model can be used to subtract the effects of brightness to determine the subjects’ emotional arousal as a function of the scene viewed.

The skin temperature signal represents changes in blood flow controlled by autonomic nervous system activity. Several studies have found that skin temperature is an effective indicator of human activity and other psychophysiological states, including affect, stress, attention, cognitive load, and drowsiness. Ioannou et al. reviewed 23 experimental procedures that used functional infrared thermal imaging to investigate this effect [32]. A major advantage of this approach is its unobtrusiveness. In particular, the temperature of the facial skin has been the subject of several studies because the face is constantly exposed and can be measured remotely using infrared thermography [32,33,34,35].

For example, [35] investigated the use of skin temperature to estimate resting blood pressure by separating acute stress variations using a multiple regression analysis. They reported that the trained model could accurately estimate resting blood pressure from facial thermal images with a root mean square error of 9.90 mmHg [35]. Another study [33] examined the utility of infrared thermal imaging for stress research. They compared thermal images to established stress indicators (heart rate, heart rate variability, finger temperature, alpha-amylase, and cortisol) in 15 participants who underwent two laboratory stress tests: the Cold Pressor Test and the Trier Social Stress Test. Their results showed that thermal imprints were sensitive to changes in both tests and that thermal imprints correlated with stress-induced mood changes, whereas established stress markers did not [33]. In a study by [34], facial skin temperature was also used. Their results showed that “skin temperature changes have both reproducible and individual response characteristics to drowsiness” [34] (p. 875). Consequently, a convolutional neural network (CNN) model was based on the distributions of facial skin temperature and trained on feature maps and individual models for each subject. The authors reported that the discrimination rate calculated using the CNN was at least 20% higher than that obtained using conventional methods.

### 2.3. Modeling Engagement with Machine Learning

Several novel approaches have been developed to directly predict engagement using machine learning methods based on physiological signals.

To this end, DeepWalk, a graph-embedding model, was developed by [36] to predict the video engagement of an ad to detect ad fraud. This model can detect fake videos and fraud patterns in videos containing well-known brands. More generally, Ref. [37] proposed an automatic approach for processing and evaluating learner engagement. They developed a prediction model for context-agnostic engagement based on the video features of the learner content and engagement signals. Research conducted by [14] on YouTube review videos (with a total duration of 600 h) identified features and patterns relevant to emotion (valence and arousal) and trustworthiness as the underlying dimensions of engagement. Several indicators of user engagement, including views, the ratio of likes to dislikes, and sentiment in comments, served as the ground truth. A study by [38] defined a set of video metrics (including video quality, time, and average percentage of videos viewed) to model the relative engagement based on 5.3 million YouTube videos. The results show that video engagement metrics are stable over time, with most of the variance explained by video context, topics, and channel information. The study also found that the time spent watching a video is a better indicator of engagement than the number of views commonly used in recent ad engagement studies.

A study by [4] examined user engagement in the context of emotional arousal (distinguishing between relaxing or exciting stimuli) during multimedia exposure and created a model based on the patterns of physiological responses of five participants to multimedia stimuli. Using machine learning, they created a predictive model of engagement based on the physiological responses to audiovisual materials. The authors performed emotional arousal classification based on affect and physiological signals from the GSR, ECG, EOG, EEG, and PPG, extracting 98 features from the five signals. Affect recognition, emotion recognition, and classification methods were used. This study found that the patterns of physiological responses to each multimedia stimulus were effective in classifying the stimulus types. The authors reported that arousal classification was achieved with 88.9% accuracy and an average recall of 83.3% for models validated using leave-one-subject-out cross-validation. Although the reported performance is high, the selected metrics of precision and accuracy are questionable because of the high class imbalance.

The advantage of combining physiological signals with machine learning is that it allows for scalable, automatic, and continuous assessment of ad engagement without being intrusive or requiring explicit user feedback. However, research on the use of machine learning and physiology to model ad engagement remains limited.

To the best of our knowledge, the only study comparable to ours is that of [39]. They developed a basic framework for assessing engagement using electroencephalography (EEG) via the Emotiv wireless headset. The EEG of 23 participants was recorded while they watched approximately 1 min commercial ad videos. Linear discriminant analysis, Linear SVM, and Radial basis SVM classifiers were used to model engagement. The authors reported an F1 score of nearly 0.7 for a binary classification of high and low values of self-reported engagement by multiple users [39].

## 3. Materials and Methods

An observational study was conducted in which the explanatory variables were participants’ physiological signals and affect, with the response variable of ad engagement defined by the existing psychometric instrument of user engagement. The following steps were performed: 1. determination of the target group of users (young adults); 2. selection of ads used in the experiments; 3. design of the experimental procedure; 4. selection of features based on physiological sensors and affective dimensions to be used later in machine learning; 5. selection of validation, evaluation, and performance metrics; 6. creation of machine learning models (classification of ad engagement); and 7. explanation of the models using SHAP.

### 3.1. Participants

Fifty young adults participated in the experiment (34 females and 16 males; age M = 21.70 and STD = 2.36). Only the heart rate signal was recorded for all 50 participants; GSR and skin temperature were recorded for 47 participants, whereas reliable eye tracking data were recorded for 33 participants. To address the challenges of missing sensor data, machine learning algorithms that can handle missing data were used to classify ad engagement.

### 3.2. Ad Selection

The video ad materials were carefully prepared. Twelve ads were selected from the YouTube streaming platform. To address the different levels of engagement, these materials were selected in consultation with three marketing specialists from The Nielsen Company. The content was in English and originally aired in the United States. A crowdsourcing study on Clickworker (https://www.clickworker.com, accessed on 12 October 2022) was conducted to determine user interactions with YouTube’s twelve video ads. Ratings of ad engagement were collected from 360 participants (ages 18–24) who answered the question “How engaging is this ad?” on a 5-point scale (none, slightly, medium, strong, very strong).

The final selection of ads was made in collaboration with Nielsen Company media experts based on several inclusion criteria: brand awareness (known vs. unknown brand) and engagement level of the ad (lower vs. higher), which were obtained from the results of the crowdsourcing study. The final selection included four ads: Dior Joy Perfume, Coca Cola, Little Baby’s Ice Cream and Waring Ice Cream Maker. Their respective YouTube channels can be found at: https://www.youtube.com/watch?v=vfOnEaaPaF4, https://www.youtube.com/watch?v=vUMQeNw2QDA, https://www.youtube.com/watch?v=erh2ngRZxs0 and https://www.youtube.com/watch?v=GJ4P6ko_aLU (all accessed on 5 October 2022).

### 3.3. Experimental Procedure

Equal ambient and viewing conditions were maintained throughout the study for all the participants. The experiment was conducted in a simulated living room, in a controlled environment that ensured consistent artificial lighting (no windows), constant temperature (air conditioning set to 24 °C), and quiet conditions. The room size was 4.0 m × 3.8 m × 2.5 m (L×W×H), and the walls were white. The lights in the room were dimmed and illuminated at 150 lux, as suggested by [40].

The experimental design involved all the participants viewing and rating all four ads assigned to them within their designated shuffle set. This was done to control for possible carryover effects of viewing one ad to the next, as the engagement triggered by the previous sequence could influence the participant’s response to the next ad. Four sets were generated, resulting in four combinations (Set1: Ad1, Ad2, Ad3, Ad4; Set2: Ad1, Ad3, Ad2, Ad4; Set3: Ad4, Ad2, Ad3, Ad1; Set4: Ad4, Ad3, Ad2, Ad1). The number of combinations was limited to four to keep the duration of the experiment manageable. Nevertheless, the ads are arranged so that no ad precedes the same ad more than once.

Next, the four sets were randomly and evenly assigned to the participants (considering age and gender), with each participant rating only one set. Within each set, the four combinations of video ads were separated by a 2 min interval to isolate any carryover effects and give participants a break if needed.

Informed consent and demographic information were obtained from all the participants. Participants were informed of the purpose of the study and given time to familiarize themselves with the environment, wearable sensors, and procedures. Physiological signals were recorded from participants throughout the experiment.

While watching the ads, the participants sat on a sofa and looked directly at the television. The ads were played on an LCD screen with a diagonal of 49 in (approximately 125 cm). The viewing distance was set to 2 m, which was consistent with the reference viewing environment for evaluating HDTV images specified in SMPTE ST 2080-3:2017 [41], as the nominal distance of the viewer from the center of the reference screen should be 3 to 3.2 frame heights. After viewing each ad, participants were asked to rate their level of ad engagement in a survey provided on a laptop computer. The study lasted an average of 45 min.

### 3.4. Psychometric Measures

The ground truth for emotional engagement in ads was measured using the User Engagement Scale-Short Form (UES-SF) [10]. It is a 12-item questionnaire covering four dimensions of engagement: Focused Attention (FA), Aesthetic Appeal (AE), Perceived Usability (PU), and Reward (RW). The dimensions were rated on a 5-point scale and the total score was calculated as an average across the selected dimensions.

The UES-SF questionnaire items were adapted to suit the context of measuring ad engagement. This is in line with the guidelines of the UES-SF, where the items from the UES-SF dimensions can be adapted to suit the task at hand [10]. In the context of ad engagement, the UES-SF measures participants’ affective (emotional) and behavioral dimensions, focusing on positive and negative affect along with aesthetic and sensory appeal, perceived usability, interest and time, and overall experience, as shown in Table 1. For example, PU is defined as “negative affect experienced as a result of the interaction and the degree of control and effort expended”, while AE is defined as “the attractiveness and visual appeal of the interface” (or, in our case, the ad) [10]. Example items for both dimensions, tailored to our case: “PU.1: I felt frustrated while watching this Ad.” and “AE.1 This Ad was attractive”.

Along with engagement ratings, participants’ affective state (valence and arousal) and tiredness were also measured using self-reports. Valence and arousal are independent, bipolar dimensions of affect, represented on a scale from pleasant to unpleasant and from active to passive, respectively. According to [42], any emotion can be described in terms of two basic dimensions. Both affective dimensions were measured on a 7-point scale (e.g., extremely passive–extremely active). Tiredness was measured on a 5-point scale (extremely tired–not tired at all).

### 3.5. Physiological Measurements

Several physiological sensor signals were recorded from the participants: eye tracking data were recorded using the Tobii Pro Glasses 2 eye tracker (pupil dilation). Heart rate, skin temperature, and electrodermal activity (EDA) were recorded using Empatica E4 wrist bracelet [43] placed on the dominant wrist.

Time synchronization of the signals was ensured by the user making a single clap before the video started. The clap was identified on the video recorded using Tobii Pro Glasses and Empatica E4 signals. Using the video and signal editor, the time stamps of all sensor devices were synchronized manually. After the synchronization, a spline signal representation of order 3 was applied, and the missing value analysis and corrected nonuniform sampling of all the time-dependent physiological signals were performed. All signals were then resampled to a common sampling frequency of 30 Hz which matched the frame rate of the video.

#### 3.5.1. Heart Rate

Raw heart rate data were acquired using the Empatica photoplethysmography (PPG) sensor, an unobtrusive method commonly used to monitor heart rate parameters and oximetry [44]. The original data were sampled at 64 Hz, filtered, and resampled at 128 Hz using a spline-based algorithm to replace missing samples and enhance peak locations. Data were processed and sampled using the Python library Neurokit2 for biomedical signal processing [45]. The PPG signal for heart rate analysis was processed using the Elgendi processing pipeline [46]. Using an interpolated sample rate of 128 Hz, we scaled down the heart rate measurement resolution based on the peak detection to below 1 bpm. The PPG-established time-varying heart rate values were interpolated using monotonic cubic interpolation and exported at 30 Hz sample rate. For segmented signal analysis, specifically for the identification of inter-beat intervals required by HRV feature extraction, the built-in capability of the library was used to process event-separated signal segments called epochs.

#### 3.5.2. Electrodermal Activity and Skin Temperature

Empatica E4 was also used to acquire electrodermal activity (EDA) and skin temperature, both at a sampling rate of 4 Hz. A spline-based algorithm was used to filter the signal and handle the missing samples. The EDA data included the number of peaks detected in skin conductance response (SCR) for each segment and the corresponding mean values of the peak amplitudes. The reported accuracy of the temperature sensor was 0.2 °C and its resolution was 0.02 °C. As only one wristband was used throughout the study, no bias adjustment was performed. The data for the EDA and skin temperature were resampled to 30 Hz to match the sample rate of the other signals.

#### 3.5.3. Pupillary Response

Pupil responses were measured using Tobii 2 eye-tracking glasses [47] and changes in pupil diameter were extracted as raw signals. Raw data were reported by the devices at nonuniform intervals. A spline-based algorithm was used to process and resample the raw pupil response data, while maintaining the mean pupil diameters of the left and right eyes. A blind luminance compensation method was used to compensate for the effects of luminance in the direction of gaze on the pupil diameter. Literature indicates that the pupillary light response to screen viewing is likely linear [31]. Therefore, for each participant, an OLS model was applied to the pupil data as a function of the display brightness throughout the observation period. The obtained gain parameter was used individually for each participant to determine the influence of brightness variance and to subtract the modeled response from the actual pupil dilation values.

### 3.6. Statistical Analysis and Machine Learning

Data preprocessing, statistical analysis, and visualization were performed in Python v.3.10 [48] using the libraries pinguoun v.0.5.3 [49], statsanalysis v0.2.3 [50], statsmodels v.0.14.0 (for logistic regression) [51], and seaborn v.0.12.2 [52]. The libraries mlxtend [53] and scikit-learn [54] were used for machine learning.

Shapiro–Wilk and Levene tests were used to test the normality and homoscedasticity of the distributions. Because the data were not normally distributed, nonparametric Mann–Whitney U and Kruskal–Wallis tests were used. The significance level was set at α = 0.05, with a Bonferroni correction for multiple comparisons. The intraclass correlation coefficient (ICC) was used to test the inter-rater agreement of the UES ratings.

#### Machine Learning

The raw physiological data were preprocessed and normalized. The type of normalization is reported where relevant. Feature generation was performed for each participant and for ad. The time-series analysis library pycatch22 [55] was used to generate features from the skin temperature and pupil dilation signals. Pycatch22 generates 22 time-series specific features describing the symbolic, temporal, and frequency domains, including the distribution shape, timing of extreme events, linear and nonlinear autocorrelation, incremental differences, and self-affine scaling (for an overview and feature definitions, see [55]). Note that, before generating the features, catch22 automatically z-normalizes the data. NeuroKit2 v.0.2.1 [45], a Python library for phyisiological signal processing, was used to process and generate heart rate variability (HRV) features from heart rate signals and to extract tonic and phasic features from GSR signals. The mean and standard deviation of the tonic and phasic GSR features were used as features for EDA.

Cross-validation was used for the training and evaluation of the machine learning models. This method uses different subsamples (k-folds) of data to train and evaluate the models by running multiple iterations and averaging the performance scores. Cross-validation ensures that the model does not overfit, as can be the case in a traditional train–test split. For example, in the case of repeated stratified cross-validation, the folds are stratified based on the target, ensuring an even distribution of target data for each fold, repeating the cross-validation procedure multiple times.

All features with collinearity > 95% and/or zero variance were removed. Further analysis and selection were performed using recursive feature elimination (RFE) with 5-fold cross-validation and the LightGBM (LGBM) classifier from scikit-learn. The most important features of each signal were retained. A total of 30 features from the four signals (HRV, EDA, skin temperature, and pupil size) were used for classification.

The effects of signal fusion on the performance of the classifier were analyzed using Exhaustive feature selector [53] by selecting and evaluating all possible signal combinations 5-fold cross-validation repeated 3 times and the LGBM. The repeated k-fold method ensures the objective validation of feature selection.

The gradient boosting classifiers LGBM, HistGradientBoostingClassifier (HGBC) and XGBoost (XGB) from scikit-learn were used as machine learning models [54]. Gradient boosting is a type of ensemble modeling technique in which multiple base models (e.g., decision trees) are trained, and the predictions of the base models are then aggregated into a single prediction by the ensemble model [56]. A gradient boosting model “is built in a stage-wise fashion as in other boosting methods, but it generalizes the other methods by allowing optimization of an arbitrary differentiable loss function” [57]. An additional advantage of gradient boosting classifiers is that they are insensitive to scale differences in data and can handle missing data. The latter is particularly relevant in cases where sensor malfunctions and recording errors could significantly decrease available training data, which is often the case in real-world settings, as is in this case.

Repeated stratified k-fold cross-validation (n_splits = 10, n_repeats = 5) was used to evaluate the classifier performance, with the ROC AUC serving as a measure of model performance. The optimization configurations for all classifiers were left at the default values.

Several steps were taken to improve the interpretability of the gradient boosting classifiers. SHAP values were used to explain the output of the classifier and the effect of each feature on the model [58]. Additionally, several baseline logistic regression models were trained on the raw signal data and selected feature sets from sensor fusion to provide further insights into the impact of features on modeling ad engagement.

## 4. Results

### 4.1. Self-Reported Measures of Engagement and Affect

Table 2 shows the correlations among the individual dimensions of the UES, namely FA, AE, PU, and RW. As shown in Table 2, there was a moderate to high correlation between FA and RW (r = 0.70 *p* < 0.001) and between AE and PU (r = 0.72, *p* < 0.001).

**Figure 1:**
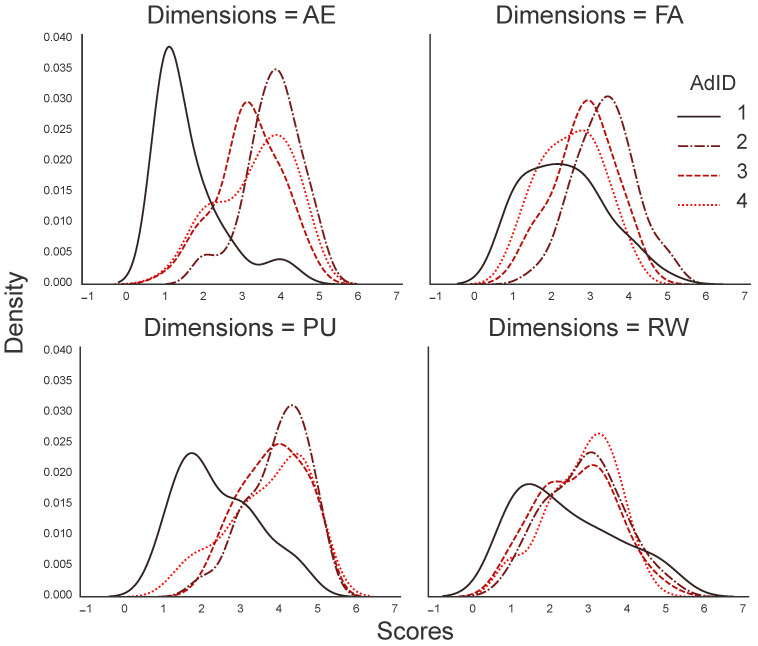
A kernel density estimate plot showing the distributions of engagement scores for each ad and individual UES dimension.

Figure 1 shows a kernel density estimate depicting the distributions of engagement scores for each ad and individual UES dimension. Differences among the four engagement dimensions were observed for each ad. The engagement in Ad1 differs from the other three ads in all the dimensions, and particularly for AE and PU.

**Figure 2:**
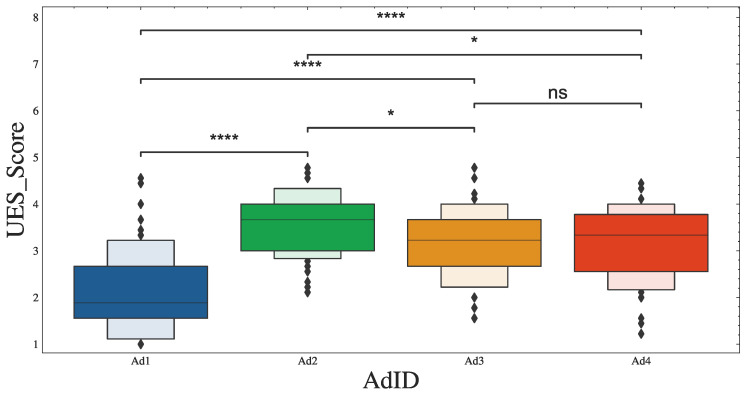
Significant differences were found in UES scores between the ads. Star notation shows *: α < 0.05, and ****: α < 0.0001, with ns = no significance.

This was confirmed by the Kruskal–Wallis test, which revealed significant differences in engagement scores among the four ads (H = 61.321, *p* < 0.001). Pairwise comparisons between the groups (with Bonferroni correction) showed the following H statistics: Ad1 vs. Ad2 (H = 47.21), Ad2 vs. Ad3 (H = 8.58), Ad3 vs. Ad4 (H = 0.03), Ad1 vs. Ad3 (H = 31.36), Ad2 vs. Ad4 (H = 7.53), and Ad1 vs. Ad4 (H = 28.65). Figure 2 shows boxplots indicating the differences in engagement scores among the ads, together with their significance levels (α statistics).

In addition, the inter-rater agreement for UES ratings of ad engagement was calculated using a 2-way mixed effects model, with an ICC(3,1) = 0.342 and a confidence interval of 95% (0.13–0.88). This poor agreement reflects the wide variation in the engagement ratings of the ads, as shown in Figure 2. It might also reflect respondent bias and differences in individual preferences regarding the advertised brand or content of the ads.

The Mann–Whitney U test showed no significant differences in the UES scores for age or gender. The Kruskal–Wallis test showed no significant differences between the ads for the four physiological signals.

Significant differences in valence and arousal were found between younger (19–22 years) and older (23–30 years) participants, but not for gender. The Mann–Whitney U test showed a significant difference between the two age groups for valence (U = 5718, α < 0.02) and arousal (U = 5786, α < 0.01). The Kruskal–Wallis tests showed no significant effect of arousal or valence on engagement scores.

### 4.2. Physiological Measures of Engagement

The Kruskal–Wallis tests also showed no significant effect of heart rate, EDA, skin temperature, or pupil dilation on the engagement scores. The means of the raw data calculated per participant and ad are listed in Table 3.

**Figure 3:**
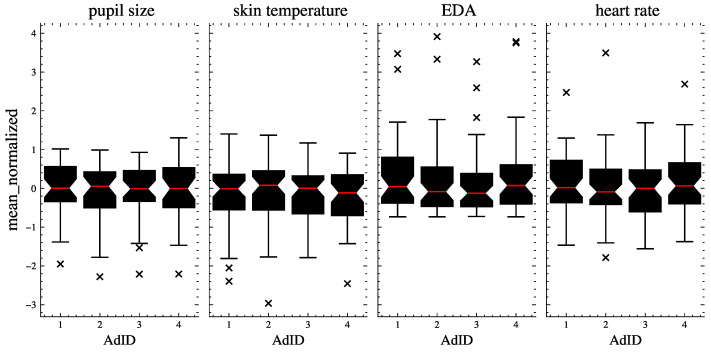
The distribution of raw signal data (means of pupil size, skin temperature, EDA, and heart rate) per signal and per ad. The red lines indicate the medians, the boxes show the quartiles, and the whiskers extend to show the rest of the distribution. The outliers are represented with ’x’.

Figure 3 shows the distribution of the signal data across the ads. The data were normalized using sklearn’s RobustScaler to account for outliers and enable a comparison of the signal data distributions on the same scale. This method removes the median and scales the data according to the interquartile range [54].

### 4.3. Classification of Ad Engagement

#### 4.3.1. Feature Selection

**Figure 4:**
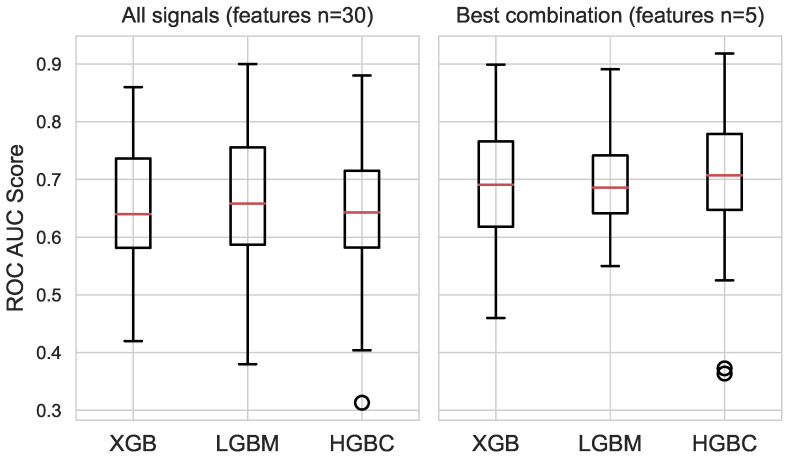
Classifier performances on the subset of features for all signals (**left**) vs. best signal combination (**right**).

Several steps were taken in the selection of features and the subsequent fusion of physiological and affective signals. First, the features with high multicollinearity and/or low variance were discarded. The most relevant features for each signal were selected using the exhaustive feature selector method with the LGBM classifier. The combined set of physiological and affective features includes 30 features, shown in Table 4. The set includes features for HRV and EDA and the time-series-specific catch22 features for pupil dilation and skin temperature. This set was used to train the three classifiers, and their performances are shown in Figure 4(left). The prefixes indicate the type of physiological signal. The catch22 feature definitions for pupil size and skin temperature statistics are referenced from [55].

#### 4.3.2. Signal Fusion

The effects of signal fusion on the classification performance were analyzed using the exhaustive feature selector method [53] by sampling and evaluating all possible signal combinations using 5-fold cross-validation and the LGBM classifier. Table 5 shows the top ten signal combinations, along with the IDs of their respective features (refer to Table 4), the average ROC AUC scores, the confidence interval bounds of the scores, their standard deviations, and their standard errors. The best-performing fusion set includes the physiological and affective signals of skin temperature, tiredness, and valence.

#### 4.3.3. Classifier Performance

**Figure 5:**
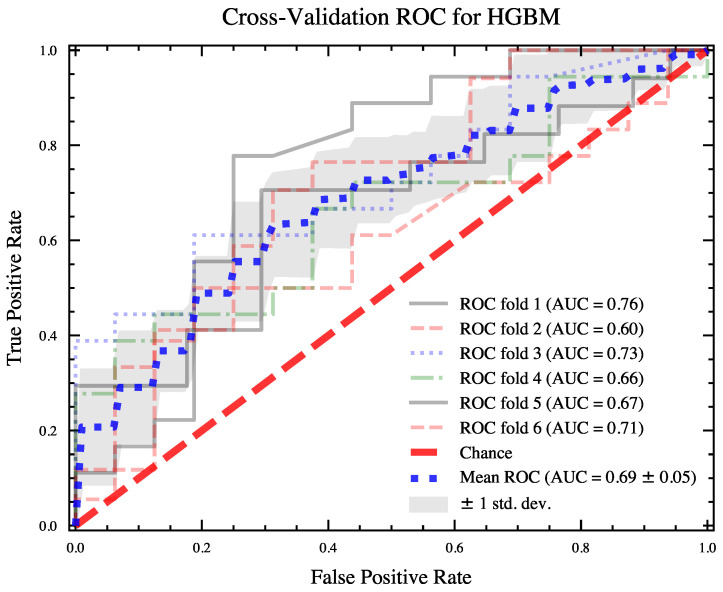
ROC curves with average AUC scores reported for each fold of 6-fold cross-validation on HGBM classifier.

Three machine learning classifiers, LGBM, XGB, and HGBC, were trained on the combined feature set and on the best-performing set of features from the signal fusion. The classifiers based on the combined feature set exhibited poorer overall performance, as shown in Figure 4(left). The average AUC ROC scores and the standard deviations of the scores for the classifiers based on the combined set (30 features) are as follows: XGB = 0.65 (0.11), LGBM = 0.66 (0.11), and HGBC = 0.64 (0.10). The classifiers trained on the fusion subset (five features) have better average AUC ROC scores: XGB = 0.68 (0.10), LGBM = 0.69 (0.07), and HGBC = 0.70 (0.11). The best performances for each classifier were obtained as follows: XGB = 0.89, LGBM = 0.89, and HGBC = 0.92. The best performing classifier was further validated by investigating the ROC curve. Figure 5 shows the ROC curves for the HGBM classifier, with respective AUC values and their averages for each fold of the 6-fold cross-validation.

### 4.4. Interpretation of the Models Using SHAP

Several steps were taken to improve the interpretability of the gradient boosting classifiers presented in the previous section. SHAP was used to interpret the global effects of the features on the classifier performance. For this purpose, a train–test split (80:20), stratified on the target variable (ad engagement), was used to train the XGB model.

**Figure 6:**
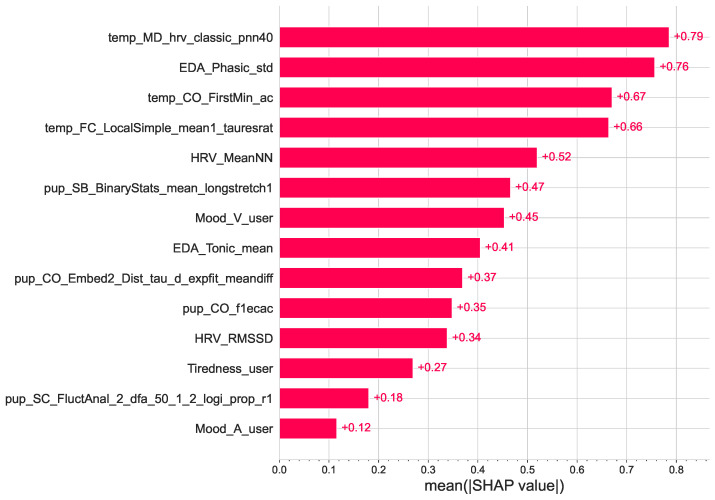
Global effect of the features on the gradient boosting classifier (XGB). Features are ranked in descending order of importance.

Figure 6 shows a global feature importance plot based on the SHAP values. The global importance of each feature is taken as the mean absolute SHAP value for that feature over all samples [58]. As shown in the figure, skin temperature features are among the best overall predictors of ad engagement.

**Figure 7:**
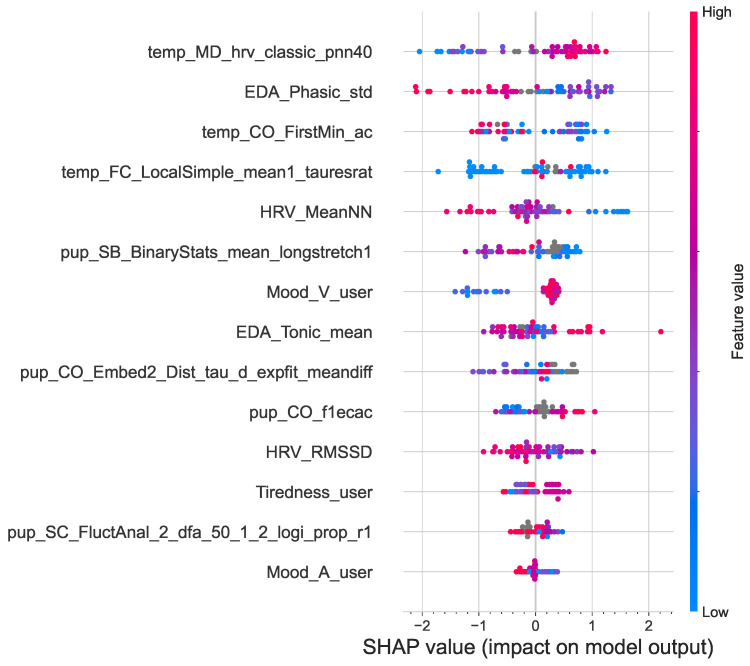
Summary plot of the positive and negative relationships of the features with ad engagement. Features are ranked in descending order of importance.

Figure 7 shows a SHAP summary plot of the impact of the features on the output of the model. The features are ranked in descending order of importance. The dots represent the instances of each feature, with the horizontal position of a dot determined by the SHAP value of that feature. The horizontal location of a dot shows the effect of its value on the prediction (lower vs. higher engagement). The density of each feature is observed from the swarm plot. Color is used to display the original value of a feature, high (red) or low (blue) [58]. For example, a high level of temp_MD_hrv_classic_pnn40 has a high positive correlation with higher ad engagement. Similarly, EDA_Phasic_std and HRV_MeanNN are negatively correlated with higher ad engagement. The results from the SHAP analysis also show that the top six most prominent features are all from the four physiological signals, confirming their potential in modeling ad engagement.

Further insights were gained through logistic regression models trained on raw signal data and selected feature sets, demonstrating the influence of specific physiological signals on ad engagement (see Appendix A). These models substantiate the results presented herein.

## 5. Discussion and Conclusions

This study investigated the potential of physiological signals and affect as reliable predictors of emotional engagement in video ads. The results presented in Section 4 and Appendix A confirm the main hypothesis that engagement in video ads can be modeled with physiological signals alone, retaining comparable performance to the models based on feature sets combining physiological signals and affect.

The key findings of this research boil down to the role of specific physiological signals and how their fusion aids in more effective modeling of ad engagement. The results also clearly show that signal fusion can significantly reduce the number of features while maintaining stable classification performance. This is particularly important in cases where continuous measurement of engagement is desired and physiological signals must be evaluated in near real time.

The three features generated from skin temperature and the two features for affect (valence and tiredness) were found to be the best performing signal fusion set. The models trained on these features also outperformed the models trained on the larger combined feature set based on all the signals. Moreover, the results presented in Table 5 show that skin temperature is an important indicator of ad engagement in several signal fusion combinations. The results of sensor fusion are further substantiated by the SHAP analysis of gradient boosting models in Section 4.4, as well as by the logistic regression models presented in the Appendix A.

To the best of our knowledge, the only comparable study using machine learning and physiological signals to investigate engagement in video ads was by [39]. Their results are similar to ours, but with a different physiology signal (EEG), a smaller number of participants (23 vs. 50), and different machine learning models and settings. The average F1 they reported is nearly 0.7 for the binary classification of high and low self-reported engagement. For general comparison, our average F1 score and its standard deviation for HGBC is 0.71 (0.06), with the best F1 score being 0.86, using repeated stratified cross-validation (n_splits = 10, n_repeats = 5).

In relation to the existing work, the significance of skin temperature as a predictor of ad engagement is a surprising finding. More prominent indicators of emotional engagement in the current state of the art are physiological signals of HRV, EDA, and pupil dilation [8,9,20,21,59,60,61].

A few studies that focused specifically on skin temperature have reported how skin temperature correlated with arousal and stress. For example, Ref. [59] conducted a study on musical emotions and found that skin temperature inversely correlated with elevated arousal and negative emotions but increased with calmness and positive emotions. A study by [60] found that the effects of stress lead to consistent temperature changes, with the temperature decreasing at distal skin locations. These findings are consistent with the results of the presented study, as shown by the SHAP analysis in Figure 7. Higher skin temperature (temp_MD _hrv_classic_pnn40) and positive valence (Mood_V_user) positively correlated with higher emotional ad engagement. In contrast, higher arousal (Mood_A_user) was negatively correlated with higher engagement, as were higher phasic EDA (EDA_Phasic_std), higher HRV (HRV_MeanNN and HRV_RMSSD), and higher pupil dilation (pup_SB _BinaryStats_mean_longstretch1).

Another observation related to existing work concerns the role of the affective dimensions of valence and arousal as indicators of ad engagement. It was expected that arousal would be a strong indicator of emotional engagement, as several studies have reported that elevated arousal increased engagement behavior (e.g., [5,7,14]). Instead, in the presented study, valence was a better predictor, which may suggest that the participants were influenced more by whether the content of the ad was pleasant than by its arousal level. The negative correlation between elevated arousal and higher ad engagement is consistent with the findings of [5], who showed that arousal is positively related to the noticeability of the ad, but can still elicit negative attitudes toward the ad. The authors argued that the negative relationship between arousal and attitude toward the ads can be explained “by creative executions of the ads, which do not appear to be positively perceived” [5] (p. 9).

In terms of limitations, the study’s reliance on self-reported measures for ad engagement could potentially introduce bias, as participants’ responses may be subject to factors such as social desirability or lack of introspective accuracy. The ICC statistical test showed low inter-rater agreement on the ad engagement rankings. Moreover, the generalizability of the findings may be limited due to the relatively small, non-random sample. In addition, this study did not consider the effects of different types of ads or product categories on physiological responses and engagement behaviors, which could significantly influence ad engagement. With more data, it might be beneficial to analyze engagement at multiple levels rather than just as a binary classification problem (lower vs. higher).

Despite these limitations, we believe that the research presented here offers new insights into the physiological measurement of ad engagement and the role of signal fusion in classification performance. Future research on this topic will aim to expand upon the findings by increasing the sample size. This research would benefit from a wider diversity of participants, which could potentially yield more varied and comprehensive results. Different physiological measures could also be explored to ascertain their effectiveness in predicting ad engagement. More importantly, the incorporation of additional variables in the classifiers, such as demographics and situational context, could contribute to improved prediction models for ad engagement.

In conclusion, the presented research contributes to the existing body of knowledge by investigating the potential of machine learning and physiological signals as predictors of ad engagement. It highlights the importance of signal fusion and demonstrates how a dimensionally reduced set of physiological signals can provide reliable classification.

## Figures and Tables

**Table 1:** The UES-SF questionnaire items [10] were adapted to measure participants’ engagement with the ads: affect, aesthetic and sensory appeal, perceived usability, interest, and overall experience in terms of reward.

Code	Item
FA-S.1	I lost myself in this experience.
FA-S.2	The time I spent watching this Ad just slipped away.
FA-S.3	I was absorbed in this experience.
PU-S.1	I felt frustrated while watching this Ad.
PU-S.2	I found this Ad confusing.
PU-S.3	Watching this Ad was taxing.
AE-S.1	This Ad was attractive.
AE-S.2	This Ad was aesthetically appealing.
AE-S.3	This Ad appealed to my senses.
RW-S.1	Watching this Ad was worthwhile.
RW-S.2	My experience was rewarding.
RW-S.3	I felt interested in this experience.

**Table 2:** Correlations between UES dimensions Focused Attention (FA), Aesthetic Appeal (AE), Perceived Usability (PU), and Reward (RW).

	FA	AE	PU	RW
FA	1	0.65	0.49	0.70
AE	0.65	1	0.72	0.58
PU	0.49	0.72	1	0.44
RW	0.70	0.58	0.44	1

**Table 3:** Descriptive statistics of physiological signals, per ad.

AdID	Type	N	Mean	SD	SE	95% Conf.	Interval
1	EDA	45	3.16	2.5	0.37	2.40	3.92
	heart rate	48	73.49	9.48	1.36	70.74	76.24
	pupil size	33	4.89	0.97	0.16	4.54	5.23
	skin temperature	45	31.66	1.55	0.23	31.19	32.13
2	EDA	47	2.78	2.65	0.38	2.01	3.56
	heart rate	49	72.53	10.47	1.49	69.52	75.54
	pupil size	33	4.76	0.99	0.17	4.40	5.11
	skin temperature	47	31.84	1.44	0.21	31.42	32.27
3	EDA	46	2.49	2.27	0.33	1.81	3.16
	heart rate	48	71.38	9.00	1.29	68.76	73.99
	pupil size	33	4.84	0.94	0.16	4.50	5.18
	skin temperature	46	31.75	1.33	0.19	31.35	32.14
4	EDA	45	2.98	2.68	0.40	2.17	3.79
	heart rate	49	74.01	9.86	1.40	71.17	76.84
	pupil size	33	4.80	0.95	0.16	4.46	5.14
	skin temperature	45	31.66	1.31	0.19	31.26	32.05

**Table 4:** Combined set of physiological and affective features (n = 30).

ID	Feature	Definition
1	HRV_MeanNN	Mean of the NN (R-R) intervals
2	HRV_RMSSD	Root mean square of successive diff
3	HRV_CVSD	RMSSD divided MeanNN
4	EDA_Phasic_mean	Mean of the phasic EDA
5	EDA_Phasic_std	Standard deviation of the phasic EDA
6	EDA_Tonic_mean	Mean of the tonic GSR
7	temp_CO_FirstMin_ac	First minimum in the autocorrelation
8	temp_CO_f1ecac	Nonlinear autocorrelation
9	temp_DN_HistogramMode_10	10-bin histogram mode
10	temp_DN_HistogramMode_5	5-bin histogram mode
11	temp_FC_LocalSimple_mean1_tauresrat	Change in autocorrelation
12	temp_FC_LocalSimple_mean3_stderr	Error of 3-point rolling mean forecast
13	temp_MD_hrv_classic_pnn40	High fluctuation
14	temp_SB_MotifThree_quantile_hh	Entropy pairs
15	temp_SC_FluctAnal_2_dfa_50_1_2	Rescaled range fluctuation analysis
16	temp_SC_FluctAnal_2_rsrangefit_50_1	Rescaled range fluctuation analysis
17	temp_mean	Mean skin temperature
18	pup_CO_Embed2_Dist_tau_d_expfit_meandiff	Embedding distance distribution
19	pup_CO_f1ecac	Nonlinear autocorrelation
20	pup_DN_HistogramMode_5	5-bin histogram mode
21	pup_DN_OutlierInclude_n_001_mdrmd	Negative outlier timing
22	pup_DN_OutlierInclude_p_001_mdrmd	Positive outlier timing
23	pup_MD_hrv_classic_pnn40	High fluctuation
24	pup_SB_BinaryStats_mean_longstretch1	Longest stretch of above-mean values
25	pup_SC_FluctAnal_2_dfa_50_1_2_logi_prop_r1	Detrended fluctuation analysis
26	pup_SC_FluctAnal_2_rsrangefit_50_1	Rescaled range fluctuation analysis
27	pup_SP_Summaries_welch_rect_area_5_1	Power in lowest 20% frequencies
28	Tiredness_user	Tiredness of the participant
29	Mood_A_user	Mood of the participant: arousal
30	Mood_V_user	Mood of the participant: valence

**Table 5:** Best performing signal combinations. The Features column refers to the ids of the features described in Table 4.

Physiological Signals	Features	AUC	CI	STD	std_err
temp, tiredness, valence	[7, 11, 13, 28, 30]	0.68	0.04	0.12	0.02
HRV, pupil	[1, 2, 18, 19, 24, 25]	0.66	0.03	0.12	0.02
HRV, EDA	[1, 2, 5, 6]	0.65	0.03	0.11	0.02
HRV, tiredness, arousal, valence	[1, 2, 28, 29, 30]	0.65	0.03	0.10	0.01
temp, pupil	[7, 13, 18, 19, 24, 16]	0.64	0.03	0.10	0.01
temp, EDA	[7, 13, 5, 6]	0.63	0.03	0.10	0.01
temp, tiredness, arousal, valence	[7, 13, 28, 29, 30]	0.63	0.03	0.11	0.02
pupil, EDA	[18, 19, 24, 26, 5, 6]	0.63	0.03	0.11	0.02
pupil, tiredness, arousal, valence	[18, 19, 24, 26, 28, 29, 30]	0.62	0.03	0.11	0.02
EDA, tiredness, arousal, valence	[5, 6, 28, 29, 30]	0.62	0.03	0.11	0.02

**Figure A1:**
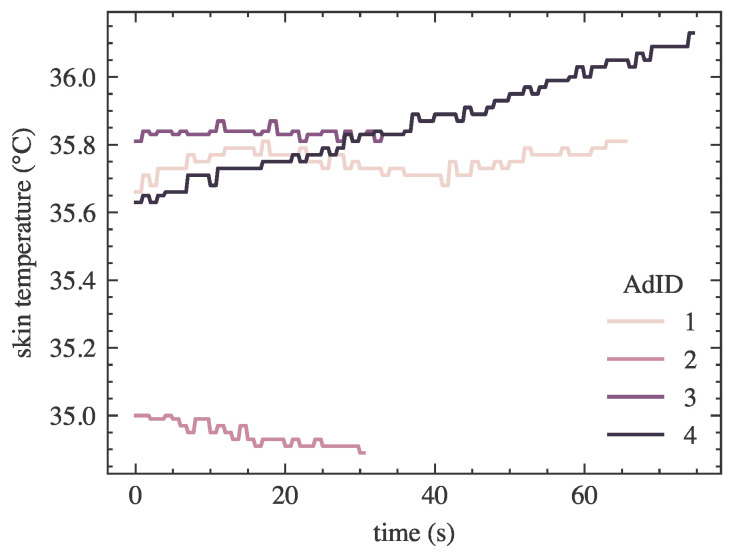
An example of a participant’s temperature course for subsequent advertisements.

## Data Availability

The data presented in this study are available upon request from the corresponding author and Supplemantary Material.

## Supplementary Materials

The following supporting information can be downloaded at: https://www.mdpi.com/article/10.3390/s23156916/s1.

## Abbreviations

The following abbreviations are used in this manuscript:

HRV	Heart Rate Variability
EDA	Electrodermal Activity
GSR	Galvanic Skin Response
catch22	Canonical Time-series Characteristics

## Appendix A. Interpretability: Baseline Logistic Regression Models

To provide further insight into the results presented in Section 4, several baseline logistic regression models were built. A baseline logit model was trained on the means of the raw signal data (calculated per person and signal) to provide insight into the signals and their impact on the target, including their marginal effects. The data were normalized using sklearn’s RobustScaler to account for outliers. This method removes the median and scales the data according to the interquartile range [54]. The results are presented below in Appendix A.1. In addition, two logit models trained on the two best performing feature sets were compared to further substantiate the results reported by the gradient boosting classifiers. The results are presented in Appendix A.2.

### Appendix A.1. Logistic Regression on Raw Signal Data

Table A1 shows the baseline logistic regression model on the raw signal data. As shown, the coefficients and z-values are highest for skin temperature (temp_mean) and heart rate (hr_mean), with standard errors being similar for all predictors.

**Table A1:** Logistic regression on raw physiological signals. Means of raw signals were used as predictors of user engagement.

Model:		Logit		Pseudo R-squared:		0.013
Dependent Variable:		UES_binary		AIC:		164.6768
Date:		2023-07-03 18:13		BIC:		178.3138
No. Observations:		113		Log-Likelihood:		−77.338
Df Model:		4		LL-Null:		−78.321
Df Residuals:		108		LLR *p*-value:		0.74209
Converged:		1.0000		Scale:		1.0000
No. Iterations:		4.0000				
	Coef.	Std. Err.	z	P > |z|	[0.025	0.975]
Intercept	0.0563	0.2064	0.2729	0.7849	−0.3483	0.4609
pup_mean	−0.0088	0.2593	−0.0340	0.9729	−0.5171	0.4995
temp_mean	0.2215	0.2452	0.9035	0.3662	−0.2590	0.7021
eda_mean	−0.1454	0.2471	−0.5883	0.5563	−0.6296	0.3389
hr_mean	−0.2346	0.2267	−1.0346	0.3009	−0.6789	0.2098

The marginal effects are presented in Table A2.

**Table A2:** Marginal effects of the logistic regression model from Table A1. The average of the marginal effects were calculated to show the impact of the predictor on the target, holding other predictors in the model constant.

Dep. Variable: Method: At:			UES_binary dydx overall			
	**dy/dx**	**Std. Err.**	**z**	**P > |z|**	**[0.025**	**0.975]**
pup_mean	−0.0022	0.064	−0.034	0.973	−0.127	0.123
temp_mean	0.0544	0.059	0.917	0.359	−0.062	0.171
eda_mean	−0.0357	0.060	−0.592	0.554	−0.154	0.083
hr_mean	−0.0576	0.055	−1.054	0.292	−0.165	0.049

### Appendix A.2. Logistic Regression for Best Performing Signal Fusion Models

This section presents the results of the logistic regression models trained on the two best performing signal fusion feature sets, as presented in Table 5 and Figure 4(right).

Table A3 shows the results of the logistic regression on the best-performing signal fusion combination, as reported in Section 4.

**Table A3:** Logistic regression on the features from the best performing signal fusion set: skin temperature, tiredness, valence (overview of model performances is in Table 5).

Model:	Logit		Pseudo R-squared:		0.061	
Dependent Variable:	UES_binary		AIC:		159.1440	
Date:	2023-07-10 17:39		BIC:		175.5084	
No. Observations:	113		Log-Likelihood:		−73.572	
Df Model:	5		LL-Null:		−78.321	
Df Residuals:	107		LLR *p*-value:		0.090762	
Converged:	1.0000		Scale:		1.0000	
No. Iterations:	7.0000					
	Coef.	Std.Err.	z	P > |z|	[0.025	0.975]
Intercept	−0.3904	0.8203	−0.4759	0.6342	−1.9981	1.2174
temp_FC...tauresrat	−0.0177	0.0088	−2.0257	0.0428	−0.0349	−0.0006
temp_CO_FirstMin_ac	−0.6753	0.4985	−1.3548	0.1755	−1.6523	0.3017
temp_MD_hrv...pnn40	0.1086	0.1373	0.7913	0.4288	−0.1604	0.3777
Tiredness	0.4177	0.8026	0.5204	0.6028	−1.1554	1.9908
Valence	0.7753	0.8932	0.8680	0.3854	−0.9754	2.5259

Table A4 shows the results of the logistic regression on the second-best performing signal fusion combination, as reported in Section 4.

**Table A4:** Logistic regression on the features from the second-best performing signal fusion set: HRV and pupil size (overview of model performances is in Table 5).

Model:	Logit		Pseudo R-squared:		0.023	
Dependent Variable:	UES_binary		AIC:		167.0375	
Date:	2023-07-08 18:22		BIC:		186.1292	
No. Observations:	113		Log-Likelihood:		−76.519	
Df Model:	6		LL-Null:		−78.321	
Df Residuals:	106		LLR *p*-value:		0.72996	
Converged:	1.0000		Scale:		1.0000	
No. Iterations:	4.0000					
	Coef.	Std.Err.	z	P > |z|	[0.025	0.975]
Intercept	−0.0295	0.2087	−0.1415	0.8874	−0.4386	0.3796
HRV_MeanNN	−0.2470	0.2319	−1.0652	0.2868	−0.7016	0.2075
HRV_RMSSD	0.0455	0.1632	0.2791	0.7802	−0.2743	0.3653
pup_CO_Embed2...	0.3170	0.2849	1.1129	0.2658	−0.2413	0.8754
pup_CO_f1ecac	−0.1960	0.3508	−0.5586	0.5764	−0.8835	0.4916
pup_SB_BinaryStats...	−0.1902	0.2683	−0.7090	0.4783	−0.7161	0.3356
pup_SC_FluctAnal_2_dfa...	−0.3121	0.3898	−0.8005	0.4234	−1.0761	0.4520

The results from the logistic regressions further substantiate the results presented in Section 4. Skin temperature has been shown to have a prominent impact on the classification of ad engagement. The reported AIC and BIC scores substantiate the best performing signal combinations presented in Table 5, with the model based on skin temperature features, tiredness and valence having lower (thus better) AIC and BIC scores as compared to the second best signal fusion model based on heart rate and pupil dilation features.

For illustration, Figure A1 shows the skin temperature changes as responses to the engagement associated with each ad, for a randomly selected participant. The lengths of the lines differ due to the different lengths of the ads.

### Appendix A.3. Logistic Regression on Affective Signals

Table A5 presents the results of the logistic regression model trained on the affective features of valence and arousal, and tiredness.

**Table A5:** Logistic regression on the affective features of valence, arousal, and tiredness.

Model:		Logit		Pseudo R-squared:		0.007
Dependent Variable:		UES_binary		AIC:		163.481
Date:		2023-07-19 13:25		BIC:		174.391
No. Observations:		113		Log-Likelihood:		−77.741
Df Model:		3		LL-Null:		−78.321
Df Residuals:		109		LLR *p*-value:		0.762
Converged:		1.000		Scale:		1.000
No. Iterations:		4.000				
	Coef.	Std.Err.	z	P > |z|	[0.025	0.975]
Intercept	−0.370	1.188	−0.312	0.755	−2.698	1.958
Valence	0.764	0.876	0.873	0.383	−0.952	2.481
Arousal	−0.421	1.096	−0.384	0.701	−2.570	1.727
Tiredness	0.270	0.904	0.298	0.766	−1.503	2.042

The AIC and BIC scores are comparable to the model based on the raw physiological signals presented in Table A1 and to the second-best performing model presented in Table A4. This further substantiates the results presented in Section 4 and confirms our hypothesis that engagement in video ads can be modeled with physiological signals alone, retaining comparable performance to the models based on the feature sets combining physiology and affect.
